# Rovibronic spectroscopy of PN from first principles†

**DOI:** 10.1039/d1cp02537f

**Published:** 2021-09-20

**Authors:** Mikhail Semenov, Nayla El-Kork, Sergei N. Yurchenko, Jonathan Tennyson

**Affiliations:** Department of Physics and Astronomy, University College London Gower Street WC1E 6BT London UK s.yurchenko@ucl.ac.uk; Department of Science and Research, Moscow Witte University 2nd Kozhukhovskiy Passage Moscow Russian Federation; Department of Physics, Khalifa University Abu Dhabi United Arab Emirates

## Abstract

We report an *ab initio* study on the rovibronic spectroscopy of the closed-shell diatomic molecule phosphorous mononitride, PN. The study considers the nine lowest electronic states, X ^1^Σ^+^, A ^1^Π, C ^1^Σ^−^, D ^1^Δ, E ^1^Σ^−^, a ^3^Σ^+^, b ^3^Π, d ^3^Δ and e ^3^Σ^−^ using high level electronic structure theory and accurate nuclear motion calculations. The *ab initio* data cover 9 potential energy, 14 spin–orbit coupling, 7 electronic angular momentum coupling, 9 electric dipole moment and 8 transition dipole moment curves. The Duo nuclear motion program is used to solve the coupled nuclear motion Schrödinger equations for these nine electronic states and to simulate rovibronic absorption spectra of ^31^P^14^N for different temperatures, which are compared to available spectroscopic studies. Lifetimes for all states are calculated and compared to previous results from the literature. The calculated lifetime of the A^1^Π state shows good agreement with an experimental value from the literature, which is an important quality indicator for the *ab initio* A–X transition dipole moment.

## Abstract 2

We report an *ab initio* study on the rovibronic spectroscopy of the closed-shell diatomic molecule phosphorous mononitride, PN.

## Graphical abstract

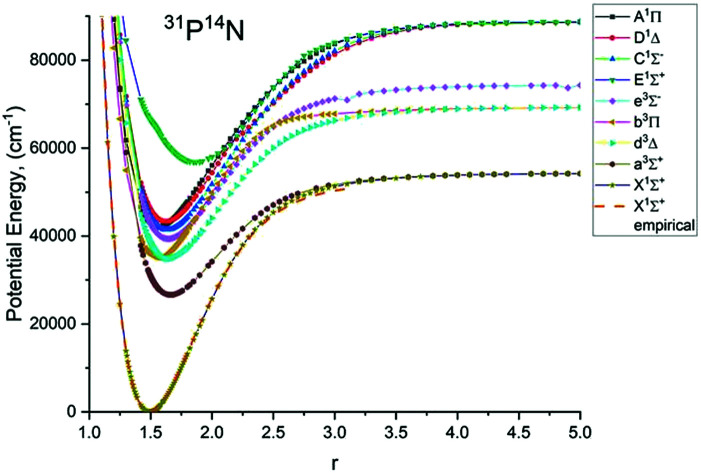

## 1 Introduction

Phosphorus is considered to be one of the key elements as a source of life and replication on our planet,^1,2^ with PN being one of the candidates in star and meteorite evolution to provide the necessary life building material. There have been multiple observations of PN in different media in space: hot dense molecular clouds,^3,4^ energetic star forming regions,^5,6^ cold cloud cores,^5,7^ red giant stars^8–10^ and protoplanetary nebula.^8^ Regardless of high astrophysical and astrobilogical importance, phosphorous mononitride is one of the experimentally least-well studied diatomic molecules of its isoelectronic group (P_2_, SiO, N_2_, CS).

The molecule was reported for the first time by Curry *et al.*^11^ using a spectroscopic study. Many other subsequent spectroscopic studies have taken place including photoelectron,^12^ fluorescence,^13^ matrix infrared,^14^ microwave^15–17^ and Fourier Transform Infra-Red (FTIR).^18^ Most of the high resolution spectroscopy experiments with PN concentrated on the electronic system A ^1^Π–X ^1^Σ^+^,^11,13,19–21^ with E ^1^Σ^+^–X ^1^Σ^+^ being confirmed afterwards,^22–24^ and several valence and Rydberg states have been studied as well.^25,26^

Measured lifetimes can be important indicators of intensities and Einstein A coefficients. So far only one experimental work reports lifetime measurements for the A ^1^Π state of PN molecule^27^ using Hanle effect, with several *ab initio* works providing computed values for PN lifetimes.^28,29^

The mass spectrometric experiment by Gingeric^30^ reports PN's dissociation energy *D*_0_ to be 6.35 ± 0.22 eV, which is lower than experimental *D*_0_ values by Huffman *et al.*^31^ and Uy *et al.*,^32^ 7.1 ± 0.05 eV and 7.57 ± 0.03 eV, respectively. In a combination of a high-level *ab initio* and microwave spectroscopy study, Cazzoli *et al.*^17^ suggested a *D*_0_ value of 6.27 eV. This is close both to the originally predicted value by Curry *et al.*^33^ and to the experimental value of Gingeric.^30^

Several theoretical investigations of PN are available in the literature. The most recent was carried out by Qin *et al.*^29^ who reported spectroscopic constants for the lowest five singlet (X ^1^Σ^+^, A ^1^Π, D ^1^Δ, C ^1^Σ^−^, 2 ^1^Π), six triplet (including a ^3^Σ^+^, b ^3^Π, d ^3^Δ, e ^3^Σ^−^) and two quintet electronic states of PN. In that paper all the states were studied at the internally contracted multi-reference configuration interaction (icMRCI) level of theory with Davidson correction (+Q). Similarly, Abbiche *et al.*^34^ reported an *ab initio* study of seven states of PN, X ^1^Σ^+^, A ^1^Π, D ^1^Δ, C ^1^Σ^−^, 2 ^1^Π, E ^1^Σ^+^, 3 ^1^Σ^+^, as well as of 10 triplet and 3 quintet electronic states. The main purpose of their study was to interpret perturbation and predissociation effects in the observed transitions.

In this work we present a comprehensive *ab initio* spectroscopic model for the nine lowest electronic states of phosphorous mononitride, X ^1^Σ^+^, A ^1^Π, C ^1^Σ^−^, D ^1^Δ, E ^1^Σ^+^, a ^3^Σ^+^, b ^3^Π, d ^3^Δ, e ^3^Σ^−^, consisting of potential energy curves (PECs), transition dipole moments curves (TDMCs), spin–orbit coupling curves (SOCs) and angular momentum coupling curves (AMCs) using the icMRCI + Q method and calculate the rovibronic energies and transition probabilities as an *ab initio* line list for PN. Producing such line lists for molecules of astrophysical significance is one of the main objectives of the ExoMol project.^35^ These curves, with some simple adjustment of the minimum energies of the PECs, are used to solve the coupled nuclear-motion Schrödinger equation with the program Duo.^36^ The spectroscopic model and *ab initio* curves are provided as part of the supplementary material. Our open source code Duo can be accessed *via*http://exomol.com/software/. The results of Duo calculation are then used to generate rovibronic spectra of PN and compare to systems previously reported in the literature.

## 2 Computational details

### 2.1 *Ab initio* calculations

Using MOLPRO 2020,^37^*ab initio* calculations were performed for nine low-lying electronic states of PN. Apart from PECs, we also computed (T)DMCs, SOCs and EAMCs. For the *ab initio* calculations we used the icMRCI method^38^ in conjunction with the effective-core-potential (ECP) method ECP10MWB (Stuttgart/Cologne) for phosphorus^39^ and aug-cc-pCV5Z^40^ basis set for nitrogen. The initial complete active space self-consistent field (CASSCF) calculation over which the configuration interaction calculations were built was for the X ^1^Σ^+^ state only. In conjunction with ECP for phosphorus, the active space was selected to be (6,2,2,0) with (1,0,0,0) closed orbitals. The state averaging set contained 96 states: 24, 24, 24 and 24 states of the singlets, triplets, pentets and septets. This level of theory will be referenced to as icMRCI + Q/ECP10MWB.

The calculations of the spin–orbit couplings were too difficult to perform for the icMRCI + Q/ECP10MWB level of theory, taking too long to complete and producing wrong data. We therefore decided to use a non-ECP level of theory for our spin–orbit calculations. To this end, we selected the *ab initio* level of theory similar to that used by Qin *et al.*^29^ with an active space of (9,3,3,0). In this case the state averaging set consisted of 11 singlet configurations (4A_1_, 2B_1_, 2B_2_ and 3A_2_). The Douglass–Kroll correction was taken into account with or without core-valence correlation. These levels of theory will be referenced to as icMRCI/aug-cc-pV5Z(-DK) and icMRCI/aug-cc-pWCV5Z(-DK), respectively, with or without DK.

**Fig. 1:**
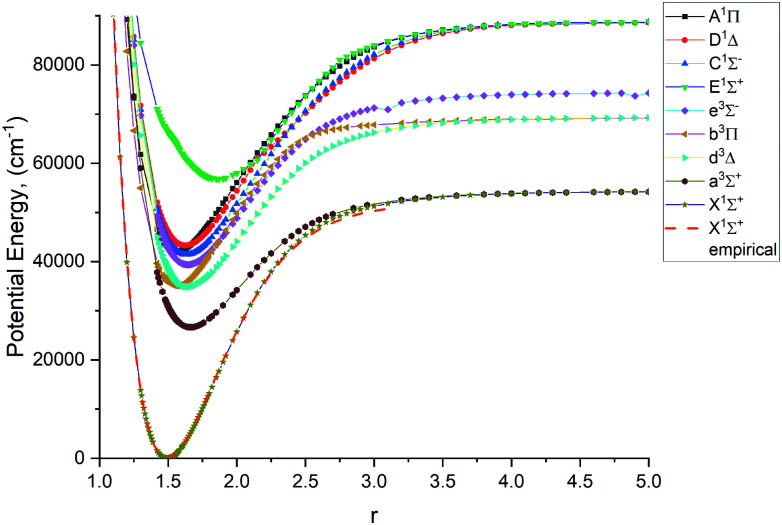
*Ab initio* (icMRCI + Q/ECP10MWB) PECs of PN: The lowest 5 singlets and 4 lowest triplets.

**Fig. 2:**
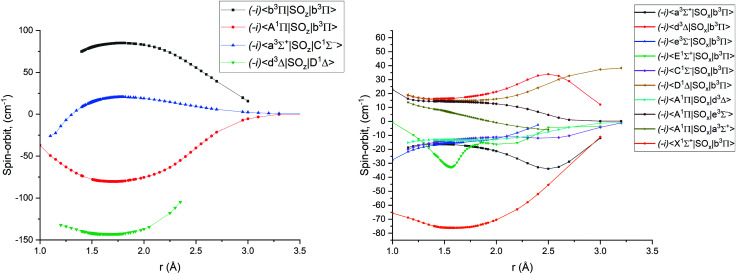
*Ab initio* spin–orbit matrix elements 〈*i*|SO_*x*_|*j*〉 for PN at the icMRCI/aug-cc-pV5Z-DK level of theory. The MOLPRO values of the magnetic quantum numbers *m*_S_ for the curves can be found in Table 2.

**Fig. 3:**
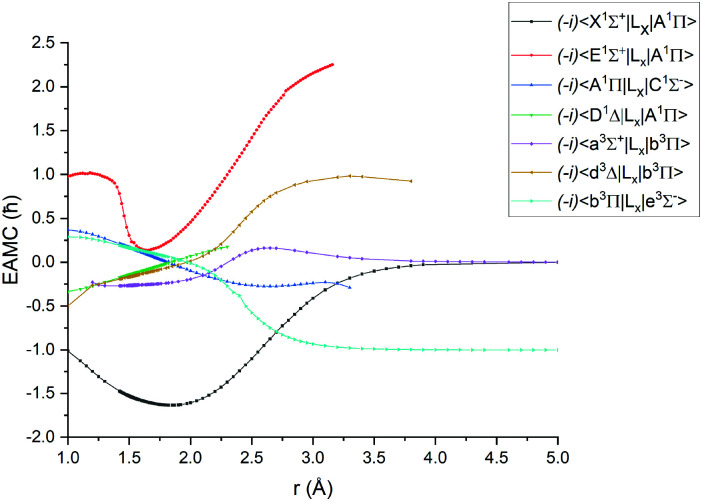
*Ab initio* (icMRCI + Q/ECP10MWB) electronic angular momentum couplings for PN in the units of *ħ*.

**Fig. 4:**
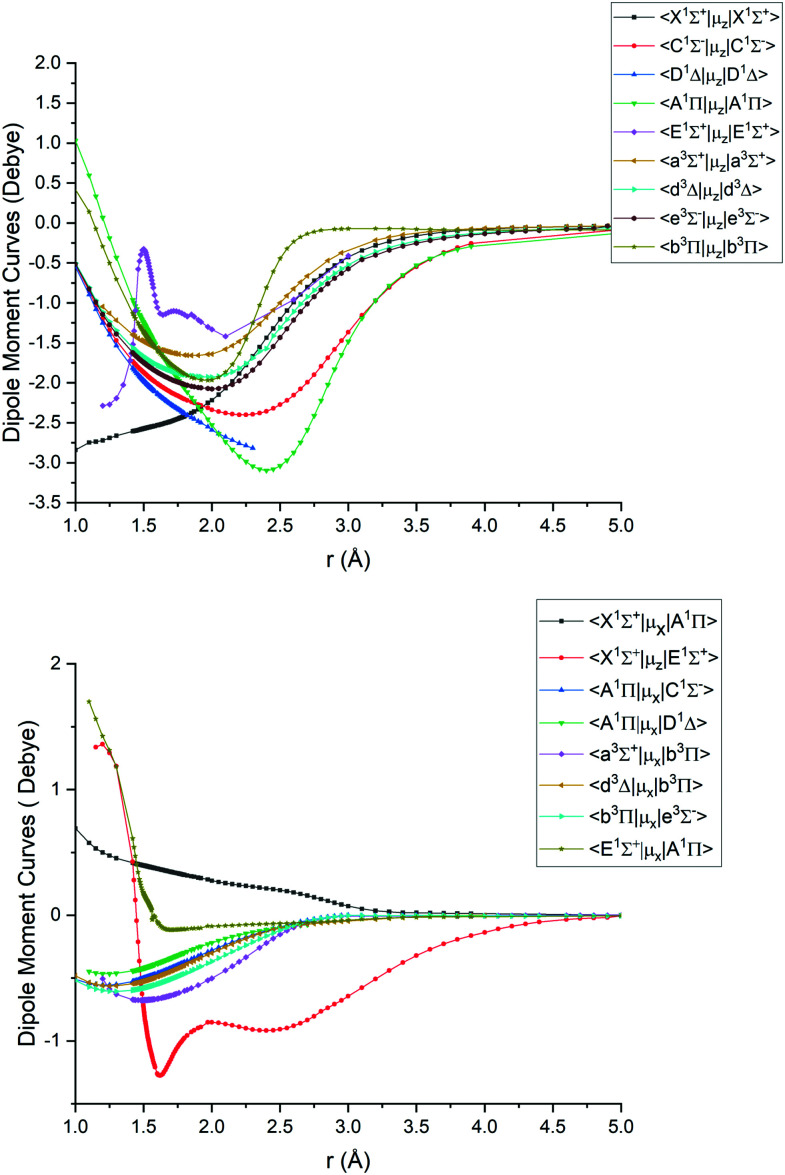
*Ab initio* (icMRCI + Q/ECP10MWB) dipole moment curves for PN: diagonal (upper), off-diagonal (lower).

Fig. 1–4 show all PECs, SOCs, EAMSc and (T)DMCs generated in this study. If the MOLPRO calculations at some geometries did not converge, they were interpolated or extrapolated from the surrounding points as part of the Duo calculations (see below). We used an adaptive *ab initio* grid consisting of 150 bond lengths ranging from 0.7 to 8 Å with more points around the equilibrium region. The grid points with the corresponding *ab initio* values (if converged) are included in the supplementary material and also shown in Fig. 1–4. The icMRCI/aug-cc-pV5Z(-DK) calculations have only converged up to about 3.5 Å due to a smaller number of configurations in the internally contacted reference set used.

### 2.2 Duo calculations

We use the program Duo^36,43^ to solve the coupled Schrödinger equation for 9 lowest electronic states of PN. Duo is a variational program capable of solving rovibronic problems for a general (open-shell) diatomic molecule with an arbitrary number of couplings, see, for example, ref. 44–47. All *ab initio* couplings between these 9 states are taken into account as described below. The goal of this paper is to build a comprehensive *ab initio* spectroscopic model for this electronic system of PN based on the icMRCI + Q/ECP10MWB and icMRCI/aug-cc-pV5Z-DK *ab initio* curves. We therefore do not attempt a systematic refinement of the *ab initio* curves by fitting to the experiment, which will be the subject of future work. In order to facilitate the comparison with the experimental data, we, however, perform some shifts of the equilibrium energy *T*_e_ and bond length *r*_e_ values, as described in further detail below.

In Duo calculations, the coupled Schrödinger equations are solved on an equidistant grid of points, in our case 501, with bond lengths *r*_*i*_ ranging from *r* = 0.85 to 5 Å using the sinc DVR method.^48^ Our *ab initio* curves are represented by sparser and less extended grids (see above). For the bond length values *r*_*i*_ overlapping with the *ab initio* ranges, the *ab initio* curves were projected onto the denser Duo grid using the cubic spline interpolation.

The following functional forms were used for extrapolation outside the original *ab initio* range:^36^*f*^short^_PEC_(*r*) = *A* + *B*/*r*,*f*^short^_TDMC_(*r*) = *Ar* + *Br*^2^,

1 *f*^short^_other_(*r*) = *A* + *Br*,

for short range and*f*^long^_PEC_(*r*) = *A* + *B*/*r*^6^*f*^long^_EAMC_(*r*) = *A* + *Br*,

2 *f*^long^_other_(*r*) = *A*/*r*^2^ + *B*/*r*^3^

for long range, where *A* and *B* are stitching parameters.

The vibrational basis set was taken as eigensolutions of the nine uncoupled 1D problems for each PEC. The corresponding basis set constructed from 9 × 501 eigenfunctions was then contracted to include about 60–80 vibrational functions from each state, enough to fill the PEC up to the corresponding dissociation energies. This gives a total of 640 states. These vibrational basis functions were then combined with the spherical harmonics for the rotational and electronic spin basis set functions. All calculations were performed for ^31^P^14^N using atomic masses.

## 3 Results and discussion

### 3.1 Results of *ab initio* calculations

The lowest 9 singlet and triplet PECs (X ^1^Σ^+^, A ^1^Π, C ^1^Σ^−^, D ^1^Δ, E ^1^Σ^+^, a ^3^Σ^+^, b ^3^Π, d ^3^Δ, e ^3^Σ^−^) calculated at the icMRCI + Q/ECP10MWB level and are shown in Fig. 1. Table 1 presents spectroscopic constants for these states estimated using the corresponding (spin–orbit-free) PECs and compares to previous studies.^17,19,29,33,34,41,42^ These values agree well for the states X ^1^Σ^+^, C ^1^Σ^−^ D ^1^Δ, E ^1^Σ^+^, a ^3^Σ^+^, e ^3^Σ^−^, b ^3^Π, d ^3^Δ as estimated using the corresponding (spin–orbit-free) PECs. For the E ^1^Σ^+^ state, however we note that there are very few studies of that state, so further computational investigation is potentially required. For the A ^1^Π state, the *T*_e_ value (the equilibrium electronic energy relative to the minimum value of X^1^Σ^+^) is by about 2300 cm^−1^ larger than previous experimental and *ab initio* values, which in turn affects the *D*_e_ value. We consider this to be an effect of the ECP method used in the *ab initio* calculations and we note that there is a similar magnitude shift in *T*_e_ of all other states.

**Table 1:** Comparison of spectroscopic constants taken from previous works and calculated from our *ab initio* curves (icMRCI + Q/ECP10MWB): Dissociation energy *D*_e_ in cm^−1^ (rounded to 3 s.f.), electronic equilibrium energy *T*_e_ in cm^−1^, equilibrium bond length *r*_e_ in Å, harmonic constant *ω* in cm^−1^, rotational constant *B*_e_ in cm^−1^

State		*D*_e_	*T*_e_	*r*_e_	*ω*_e_	*B*_e_
X^1^Σ^+^	This work	53100.00	0.00	1.49	1303.23	0.78
	Expt.^33^	50812.91	0.00	1.4869	1337.24	0.78549
	Expt.^17^	51938.86	0.00		1336.992	0.78648
	Calc^29^	51454.07	0.00	1.4918	1339.61	0.78549
	Calc.^41^	50645.15	0.00	1.4948	1333.84	0.7823
	Calc.^34^	49683.73	0.00	1.4977	1328.21	0.77872
A^1^Π	This work	44900.00	42143.66	1.56	1041.07	0.71
	Expt.^33^	41134.26	39688.52	1.5424	1103.09	0.73071
	Expt.^19^		39805.90		1103	0.731
	Calc^29^	40995.53	40032.55	1.5476	1104.81	0.7306
	Calc.^41^	41331.86		1.5501	1100.24	0.727505
	Calc.^34^	40624.26	40610.00	1.556	1078.42	0.7213
D^1^Δ	This work	43100.07	43287.03	1.63	1016.44	0.66
	Calc.^29^	39993.49	42106.86	1.6073	1018.98	0.67724
	Calc.^42^	33713.96	41618.19	1.622	967.9	0.664
	Calc.^34^	39448.89	41835.96	1.614	1010.647	0.671
	Calc.^41^	41555.28		1.6196	1002.269	0.665056
C^1^Σ^−^	This work	45300.00	41417.18	1.63	998.65	0.66
	Calc.^29^	42766.02	39298.72	1.6211	977.5	0.6639
	Calc.^42^	37827.38	37504.77	1.617	1108.8	0.669
	Calc.^34^	41852.09	39505.02	1.627	973.927	0.6597
E^1^Σ^+^	This work	30200.00	56793.82	1.86	702.48	0.50
	Calc.^34^	26188.80	55103.80	1.889	743.74	0.4893
a^3^Σ^+^	This work	26600.62	26632.70	1.66	917.51	0.63
	Calc.^29^	25249.32	26065.66	1.6481	941.61	0.64253
	Calc.^42^	19599.26	24761.21	1.669	787.4	0.628
	Calc.^34^	23785.28	25817.80	1.655	913.765	0.6375
e^3^Σ^−^	This work	32670.00	39370.41	1.64	974.15	0.65
	Calc.^29^	32081.01	37951.02	1.624	981.37	0.66149
	Calc.^42^	37827.39	37504.77	1.617	1108.8	0.669
	Calc.^34^	30665.19	37956.44	1.632	982.255	0.6556
d^3^Δ	This work	33300.00	34860.41	1.65	960.01	0.65
	Calc.^29^	29679.37	32975.56	1.6324	949.99	0.65376
	Calc.^42^	23551.38	32504.13	1.666	770.3	0.629
	Calc.^34^	28471.36	33125.18	1.64	959.263	0.6495
b^3^Π	This work	33400.00	35146.16	1.56	1070.81	0.71
	Calc.^29^	29197.77	33671.79	1.5449	1113.24	0.73301
	Calc.^42^	21696.31	34359.20	1.558	1124.3	0.72
	Calc.^34^	27672.87	33843.01	1.555	1102.494	0.7224

**Table 2:** MOLPRO magnetic quantum numbers *m*_S_ values for the *ab initio* spin–orbit matrix elements displayed in Fig. 2

SOC	Bra *m*_S_	Ket *m*_S_
〈A^1^Π_*y*_|SO_*x*_|d^3^Δ_*xy*_〉	0	1
〈A^1^Π_*y*_|SO_*x*_|e^3^Σ^−^〉	0	1
〈a^3^Σ^+^|SO_*x*_|b^3^Π_*y*_〉	0	1
〈a^3^Σ^+^|SO_*x*_|A^1^Π_*y*_〉	1	0
〈d^3^Δ_*xy*_|SO_*x*_|b^3^Π_*y*_〉	0	1
〈e^3^Σ^−^|SO_*x*_|b^3^Π_*x*_〉	0	1
〈X^1^Σ^+^|SO_*x*_|b^3^Π_*y*_〉	0	1
〈E^1^Σ^+^|SO_*x*_|b^3^Π_*y*_〉	0	1
〈C^1^Σ^−^|SO_*x*_|b^3^Π_*y*_〉	0	1
〈D^1^Δ_*z*_|SO_*x*_|b^3^Π_*x*_〉	0	1
〈b^3^Π_*x*_|SO_*z*_|b^3^Π_*x*_〉	1	1
〈A^1^Π_*x*_|SO_*z*_|b^3^Π_*x*_〉	0	0
〈a^3^Σ^+^|SO_*z*_|C^1^Σ^−^〉	0	0
〈d^3^Δ_*z*_|SO_*z*_|D^1^Δ_*z*_〉	0	0

The *ab initio* SOCs, EAMCs and (T)DMCs are shown in Fig. 2–4 respectively, with their phases indicated and the reference distance for phases taken as 1.5 Å. For all these curves the post-processing of *ab initio* calculations included (i) inter- and extrapolations for the missing points using neighboring geometries and (ii) phase mapping using a procedure similar to the one described by Patrascu *et al.*^44^ Some of the curves also cover only part of the range, which can be seen with the TDMCs 〈X ^1^Σ^+^|*μ*_*z*_|E ^1^Σ^+^〉 in Fig. 4. The rest of the points portrayed incoherent behaviour and hence were dropped in the post-processing. When calculating spectra for curves with dropped points, the lines were extrapolated using cubic splines as part of the Duo calculations.

### 3.2 The A ^1^Π–X ^1^Σ^+^ band using different levels of *ab initio* theory

**Fig. 5:**
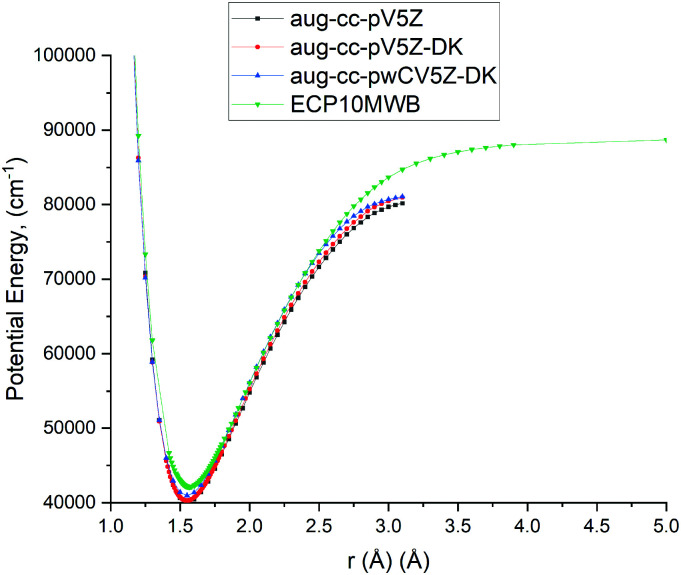
Comparison of PECs for the A ^1^Π state of PN at different levels of theory.

**Fig. 6:**
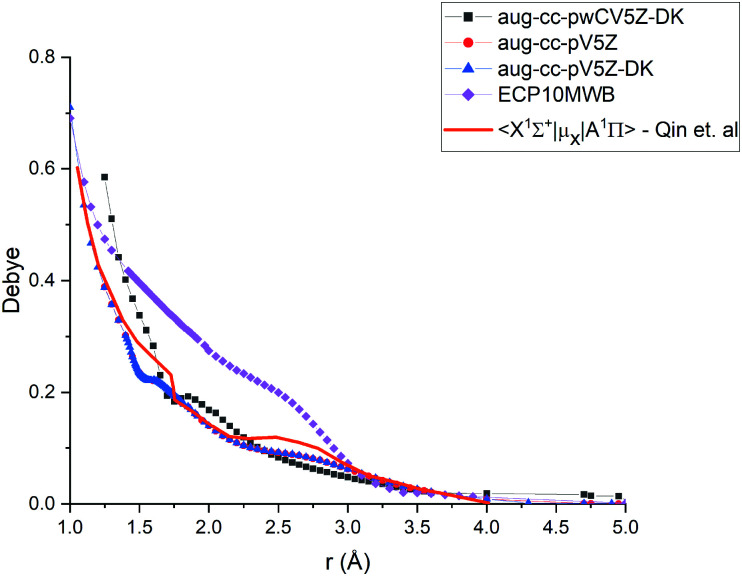
Comparison of transition dipole moment between A ^1^Π and X ^1^Σ^+^ states of PN at the different levels of theory (ECP10MWB, icMRCI/aug-cc-pV5Z-DK, icMRCI/aug-cc-pWCV5Z-DK) and previously reported results.^29^

As the A ^1^Π–X ^1^Σ^+^ band is one of the most important spectroscopic systems of PN, here we compare the X ^1^Σ^+^ and A ^1^Π PECs and the A ^1^Π–X ^1^Σ^+^ TDMC computed using different levels of theory: icMRCI + Q/ECP10MWB, icMRCI/aug-cc-pV5Z-DK and icMRCI/aug-cc-pwCV5Z-DK, see Fig. 5 and 6. As part of the spectroscopic model for PN, even a slight change of these curves can have a profound effect on the simulated spectra or lifetimes.

In Fig. 6, we present a comparison of TDMCs of the A^1^Π and X^1^Σ^+^ states calculated at different levels of theory and results previously calculated by Qin *et al.*^29^ The TDMC calculated with the ECP10MWB stands out the most with larger values of the dipole moment around the equilibrium (*i.e.* in the spectroscopically relevant region) and the rest of the ECP-free methods giving curves which lie close to each other. We show below that the ECP10MWB DMC has improved the A–X lifetime of PN, the only known experimental evidence of the transition probability of this system.

The aug-cc-pWCV5Z-DK and previous results by Qin *et al.*^29^ show smaller values of TDMC which will lead to weaker intensities and longer lifetimes.

### 3.3 Results of Duo calculations

In this study we work directly with the *ab initio* data in the grid representation without representing the curves analytically, with exception of the X ^1^Σ^+^ state for which we use an empirical potential energy function from Yorke *et al.*^49^ Using their PEC, we essentially reproduce the X ^1^Σ^+^ states ro-vibrational energies of the YYLT line list.

**Fig. 7:**
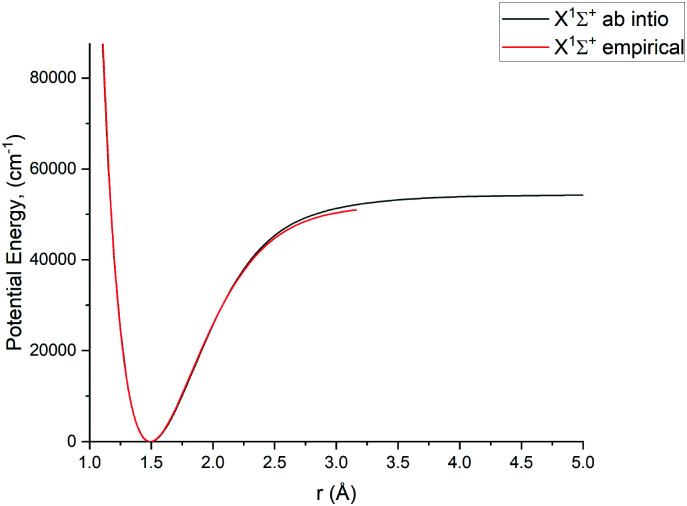
Comparison between a pseudopotential *ab initio* X ^1^Σ^+^ PEC and previously calculated empirical PEC^49^ of PN.

**Fig. 8:**
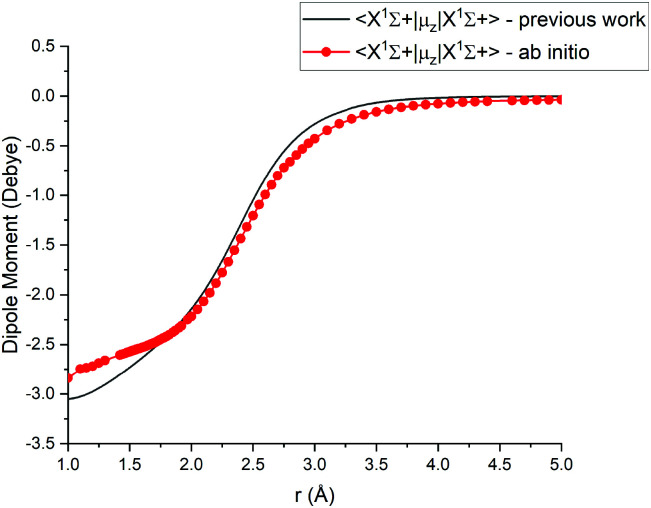
Comparison between absolute values of the *ab initio* X^1^Σ^+^ DMCs from this work (icMRCI + Q/ECP10MWB) and by^49^ (MRCI + Q/aug-cc-pCV6Z).

For Duo calculations, we selected the following set of curves: the icMRCI + Q/ECP10MWB PECs shown in Fig. 1 for all but the X ^1^Σ^+^ state. The comparison between the X ^1^Σ^+^*ab initio* PEC from this work and the empirical PEC from^49^ can be seen in Fig. 7. Similarly for (T)DMCs all curves are *ab initio*, see Fig. 4, apart from the ground state dipole moment 〈X ^1^Σ^+^|*μ*_*z*_|X ^1^Σ^+^〉, which was also taken from Yorke *et al.*^49^ The comparison between two DMCs is shown in Fig. 8. For SOCs, TDMCs and EAMCs we use *ab initio* obtained curves shown in Fig. 2–4.

The rovibronic wave functions of PN were computed with Duo and then used in conjunction with the *ab initio* (T)DMCs to produce Einstein *A* coefficients for all rovibronic transitions between states considered in this work, for the wavenumber range from 0 to 88 000 cm^−1^ and *J* ≤ 270. In these calculations, the lower states were capped by the energy threshold 60 000 cm^−1^, which is close to the lowest dissociation threshold, while the upper state threshold was set to 88 000 cm^−1^ (highest asymptotic channel of our nine electronic state system). The line list contains 233 418 251 transitions between 390 368 rovibronic states.

These Einstein *A* coefficients, organised as per the ExoMol format^50^ in a line list, were then used to calculate lifetimes and spectra. The aforementioned format uses a two file system to represent relevant spectroscopic information, with the energies and specific state quantum numbers included into the States file (.states) and Einstein coefficients appearing in the Transitions file linking different states (.trans). The.states and.trans files produced by Duo are used in conjunction with Exocross^51^ to produce spectra and lifetimes (see below).

### 3.4 Partition function

**Fig. 9:**
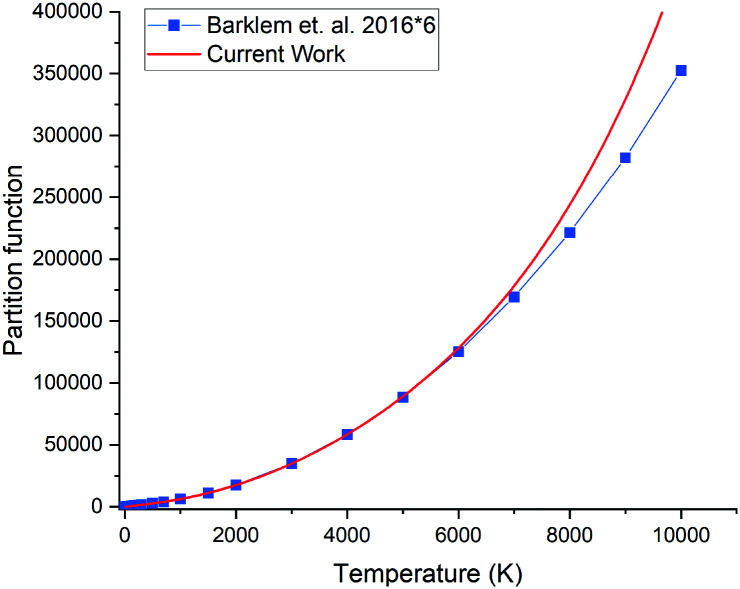
Comparison of partition functions for ^31^P^14^N: *ab initio* this work and the values of Barklem and Collet.^52^

The partition function of ^31^P^14^N computed using our *ab initio* line list is shown in Fig. 9, which is compared to that recently reported by Barklem and Collet.^52^ Since ^14^N has a nuclear spin degeneracy of 3 and ^31^P has nuclear spin degeneracy of 2, we have multiplied Barklem and Collet's partition function by a factor of six to compensate for the different conventions used; we follow ExoMol and HITRAN^53^ and include the full nuclear spin in our partition functions. The differences in higher temperatures can be attributed to incompleteness in the model used by Barklem and Collet.^52^

### 3.5 Spectral comparisons

Using the *ab initio*^31^P^14^N line list, spectral simulations were performed with our code ExoCross.^51^ ExoCross is an open source Fortran 2003 code, see http://exomol.com/software/ or https://github.com/exomol, whose primary use is to produce spectra of molecules at different temperatures and pressures in the form of cross sections using molecular line lists as input. Amongst other features, ExoCross can generate spectra for non-local thermal equilibrium conditions characterized with different vibrational and rotational temperatures, lifetimes, Landé *g*-factors, partition and cooling functions.

**Fig. 10:**
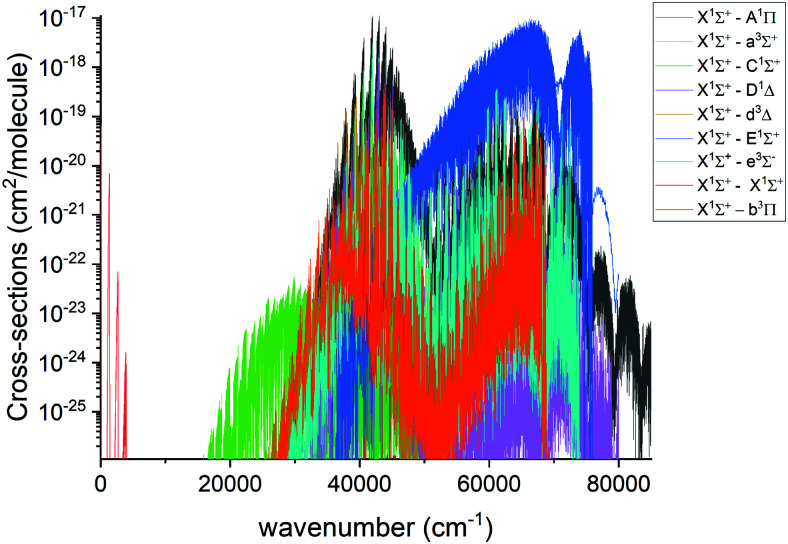
Overview of the calculated absorption spectra of PN from our model at *T* = 2000 K. A Gaussian line profile of HWHM = 1 cm^−1^ is used.

An overview of the PN absorption spectra in the form of cross sections at the temperature *T* = 2000 K is illustrated in Fig. 10. Here, a Gaussian line profile with a half-width-at-half-maximum (HWHM) of 1 cm^−1^ was used. This figure shows contributions from each electronic band originating from the ground electronic state. The only band systems that have so far been characterized experimentally are X ^1^Σ^+^–X ^1^Σ^+^ A ^1^Π–X ^1^Σ^+^, E ^1^Σ^+^–X ^1^Σ^+^. Here we mainly concentrate on discussing these band systems, due to the lack of experimental detection for other bands calculated in this work.

#### 3.5.1 X ^1^Σ^+^ band

**Fig. 11:**
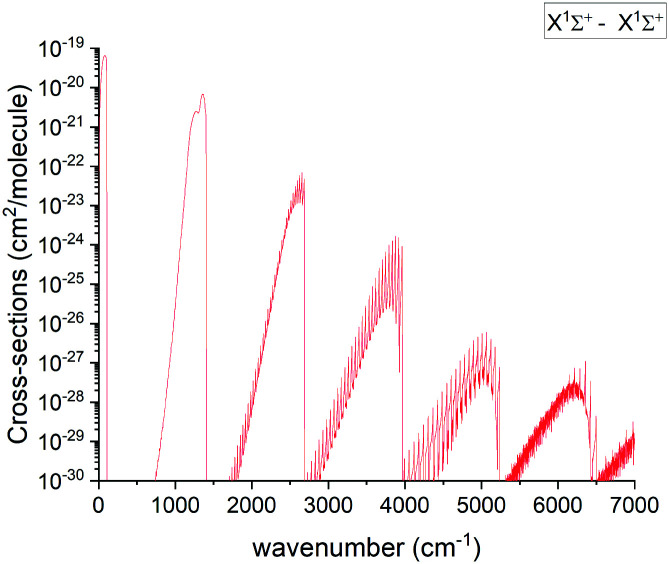
Overview of the calculated X ^1^Σ^+^ state rotation-vibration absorption spectrum of PN at *T* = 2000 K with a Gaussian line profile of HWHM = 1 cm^−1^.

The pure rotational X state transitions represent the main source of the PN observations in interstellar-medium and stellar spectra.^3–10^ Apart from astrophysical observations, the band has been analysed in multiple lab experiments as well.^14–16,18^ An overview of the calculated absorption band of PN at 2000 K is shown in Fig. 11.

#### 3.5.2 A ^1^Π–X ^1^Σ^+^ band

**Fig. 12:**
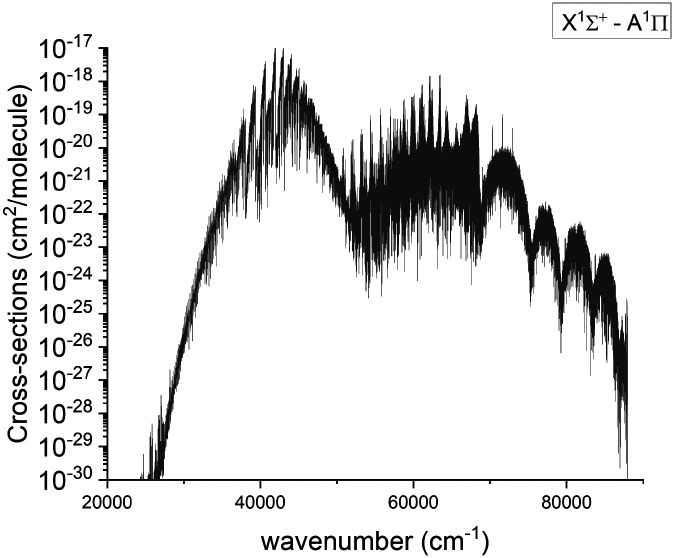
Calculated absorption spectra of the A ^1^Π–X ^1^Σ^+^ system at 2000 K with a Gaussian profile of HWHM = 1 cm^−1^.

The visible A–X band system was first observed by Curry *et al.*^33^ and there have been several subsequent laboratory observations.^13,19,21,54^ Below we compare our results to the experimental spectra; an overview spectrum for the A ^1^Π–X ^1^Σ^+^ band system at 2000 K is given in Fig. 12.

**Fig. 13:**
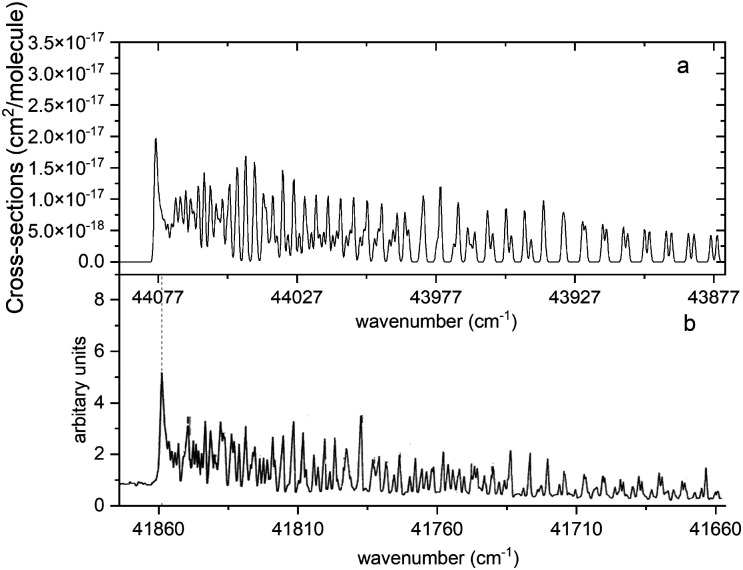
Comparison of the A ^1^Π–X ^1^Σ^+^ (2,0) simulated absorption band (panel a) with that recorded by Le Floch *et al.*^21^ (panel b). The spectrum was simulated at *T* = 1173.15 K with a Gaussian profile of HWHM = 0.5 cm^−1^ and is offset by 2217 cm^−1^ so that the features align.

Fig. 13 compares a Duo generated spectrum of the A ^1^Π–X ^1^Σ^+^ (2,0) band to a part of the spectrum from recent experimental observations by Le Floch *et al.*^21^ Even at such a high resolution the main trends of the spectrum are reproduced. However, we have to note a shift of −2217 cm^−1^ and some differences in intensities. This will be corrected with a further improvement of our model by fitting the A ^1^Π PEC to the experimental data.

**Fig. 14:**
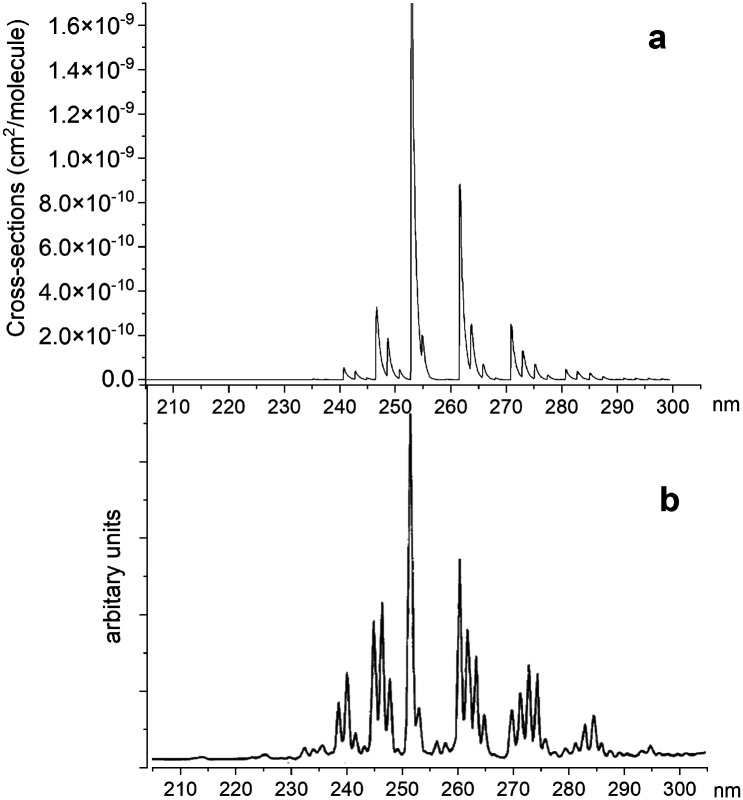
Comparison of A ^1^Π–X ^1^Σ^+^ simulated emission spectra (panel a) with that recorded by Saraswathy and Krishnamurty.^54^ (panel b). The spectra was simulated at 2000 K rotational temperature with a Gaussian profile and HWHM = 100 cm^−1^.

Fig. 14 illustrates a simulated emission A ^1^Π–X ^1^Σ^+^ band system at 250 nm, which is compared to the chemiluminescent experimental spectrum of Saraswathy and Krishnamurty,^54^ where we used the rotational temperature setting as 2000 K. However, when trying to adjust this spectrum, it proved to be very sensitive to the *T*_e_ and *r*_e_ values for the A ^1^Π state. Therefore, for the purpose of this illustration we shifted the A ^1^Π PEC to match experimental values of *T*_e_ and *r*_e_. There is also a difference in relative magnitudes, which is attributed to the difference in vibrational population distribution of the experiment and our LTE calculations, as there are non-LTE effects which contribute to the spectrum. Even though the PEC was adjusted to the experimental values previously reported by Ghosh *et al.*^19^ to reproduce this spectrum, there is still a difference of 0.6 nm between *ab initio* spectrum and experiment, which we aim to resolve with further improvements of our spectroscopic model *via* empirical refinements.

**Fig. 15:**
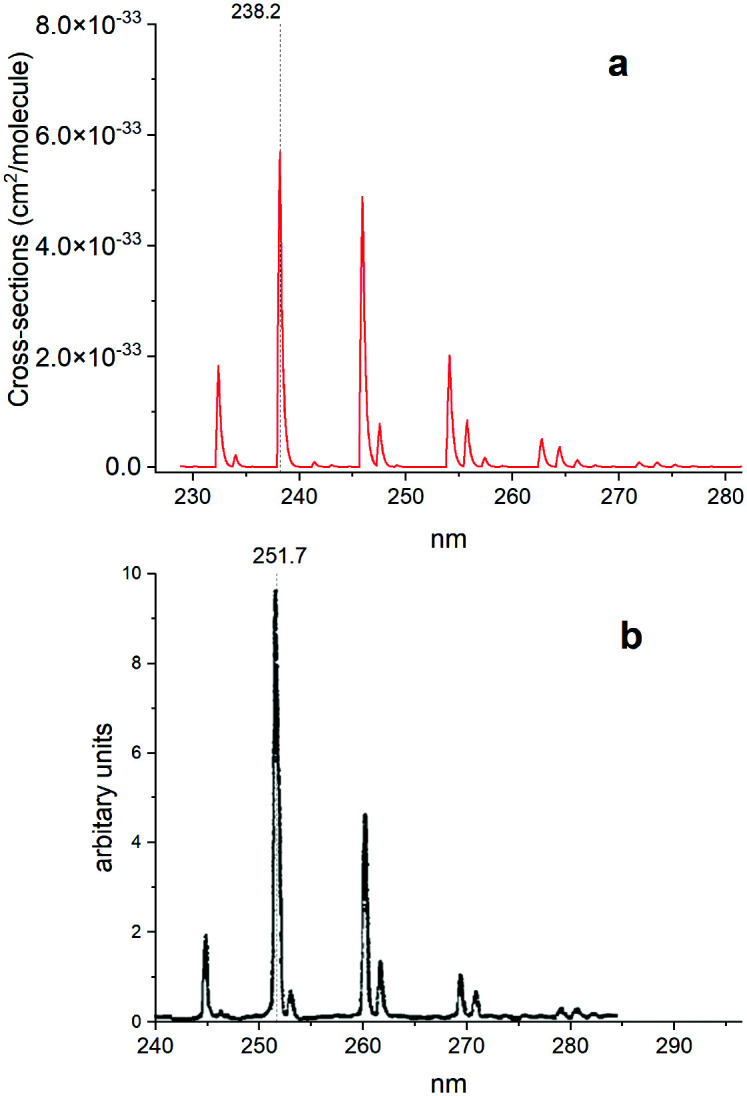
Comparison of an A ^1^Π–X ^1^Σ^+^ simulated emission spectrum (panel a) with that recorded by Moeller and Silvers.^13^ (panel b). The number at the top indicates the centre of the peak. The spectrum was simulated at *T*_rot_ = 500 K (rotational) and *T*_vib_ = 1200 K (vibrational) temperatures with a Gaussian profile of HWHM = 100 cm^−1^.

Fig. 15 shows a simulated non-LTE absorption A ^1^Π–X ^1^Σ^+^ spectrum of PN to compare to the experiment of Moeller and Silvers.^13^ Most of the major features are again shifted by ∼13.5 nm. We attribute the relative difference in strength of peaks between theory and experiment to difficulty in reproducing the non-LTE behaviour of the experiment, due to the lack of necessary information in the original paper.

### 3.6 Lifetimes

**Fig. 16:**
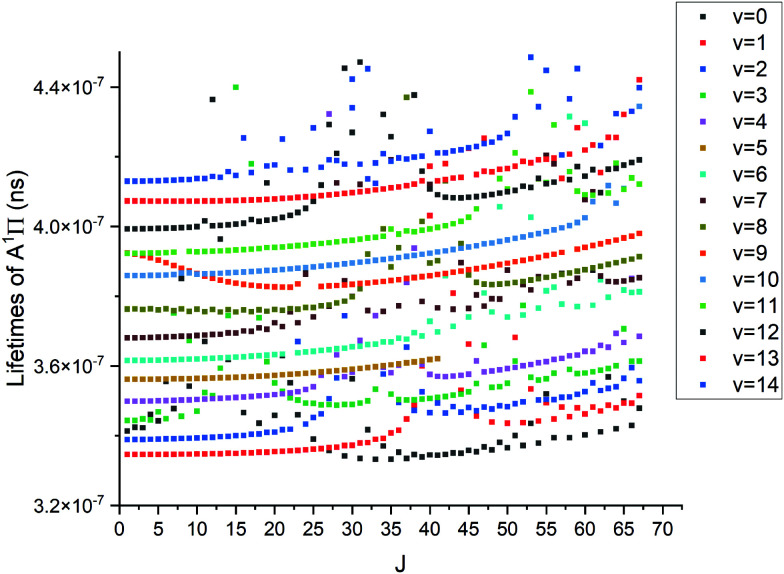
Lifetimes of A ^1^Π state *vs. J*. The top row of points is *v* = 14 whereas the bottom is *v* = 0.

In this work, lifetimes of PN are calculated using ExoCross,^51^ based on the states and transitions files generated by Duo. ExoCross calculates lifetimes as follows:3
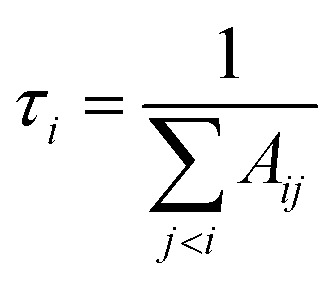
where *τ*_*i*_ is radiative lifetime, *A*_*ij*_ is Einsteins A coefficients, and *i* and *j* stand for upper and lower states, respectively. While the lifetimes reported in other works cited below are for vibrational levels and the above formula is for an individual state; however we have found no strong *J*-dependency for the PN lifetimes. This means that *J* = 0 does indeed give a good approximation for the vibrational state lifetime. The lack of this dependency can be seen in Fig. 16. The methodology used is described in detail by Tennyson *et al.*^55^Fig. 16 shows structures for different vibrational levels, which speaks to the perturbed nature of the A ^1^Π state.

The lifetimes of ^31^P^14^N in the *A*^1^Π state for (*v* = 0) were measured by Moeller *et al.*,^27^ and also were calculated by Qin *et al.*^29^ and de Brouckere *et al.*^28^ While Moeller *et al.*^27^ report a lifetime of 227 ± 70 ns for *ν*′ = 0 of A ^1^Π our value is only slightly higher at 341 ns, which is closer than previous theoretical calculations of 695.4 ns^29^ and 742.4 ns^28^ by a factor of 2. Table 3 provides a more detailed comparison between our calculated lifetimes and those of Qin *et al.*^29^ We assume that the major differences in the A ^1^Π lifetimes and previous calculation to the difference in the transition dipole moment between X ^1^Σ^+^ and A ^1^Π comparison of which can be seen in Fig. 6. In order to check that, we have extracted the A ^1^Π TDMC data from the original source^29^ and rerun the lifetime calculation, then getting a comparable result of 677 ns for *ν*′ = 0. For the b ^3^Π state, lifetimes seem to be in agreement with Qin *et al.*^29^ for all but *v* = 0, but same cannot be said for the D ^1^Δ and e ^3^Σ^−^ states. We attribute the bulk of differences in lifetimes for these states to the differences in TDMCs.

**Table 3:** Comparison of lifetimes from our current work (A) and Qin *et al.*^29^ (B). An experimental lifetime 227 ± 70 ns was reported by Moeller *et al.*^27^ for *ν*′ = 0 of A^1^Π

*ν*′	A ^1^Π/ns		D ^1^Δ/μs		b ^3^Π/μs		e ^3^Σ^−^/μs	
	A	B	A	B	A	B	A	B
0	341.3	659.4	936.03	3872	18.76	49.2	128.51	206.9
1	334.6	674.3	301.51	1186	15.38	27.81	52.43	98.46
2	338.9	660.8	120.96	615.2	13.07	19.5	73.08	59.45
3	344.3	646.5	110.39	378.3	11.43	15.05	61.85	40.75
4	349.8	642.2	88.39	246.8	10.22	12.4	53.48	30.41
5	356.2	643	69.23	181.8	10.06	10.62	47.30	23.68
6	361.6	632.1	56.85	144.3	8.55	9.364	42.60	19.26
7	368.1	610.6	47.37	113	7.94	8.356	38.88	16.16
8	376.3	607.4	40.67	9.37	7.45	7.458	35.84	13.93
9	392.4	598.6	35.52	9.85	7.04	6.881	33.40	12.2
10	399.3	583.8	31.58	10.31	6.70	6.232	31.33	10.77
11	407.3	581.8	28.33	11.32	6.42	5.592	29.60	9.517
12	413.0	566.8	25.69	13.06	6.19	5.283	28.07	8.146
13	420.7	565.3	23.34	17.18	5.99	4.819	26.78	7.142
14	431.3	553.1	21.66	22.57	5.82	4.303	25.67	6.192

Lifetimes for other singlet and triplet states are reported in Table 4. From there we can see that the radiative lifetimes are in milliseconds for C ^1^Σ^−^, d ^3^Δ and a ^3^Σ^+^, and nanoseconds for E ^1^Σ^+^. Long lifetimes for the C ^1^Σ^−^, d ^3^Δ and a ^3^Σ^+^ states are an indication of low probability of transition from these states, leading us to believe that these states would be very difficult to observe in an experiment. This is confirmed with current experimental evidence for PN, as C ^1^Σ^−^, D ^1^Δ, a ^3^Σ^+^, d ^3^Δ, e ^3^Σ^−^, which have only been observed indirectly through perturbation with the state A ^1^Π by Le Floch *et al.*^21^ The b ^3^Π, d ^3^Δ, e ^3^Σ^−^ states have been also previously identified only through perturbation with A^1^Π state by Saraswathy and Krishnamurty.^20^

**Table 4:** Calculated lifetimes for the C ^1^Σ^−^, a ^3^Σ^+^ and d ^3^Δ E ^1^Σ^+^ states with *ν*′ up to 14. A full list of lifetimes is included in the Exomol states file

*ν*′	C ^1^Σ^−^/ms	a ^3^Σ^+^/s	d ^3^Δ/ms	E ^1^Σ^+^/ns
0	65.02	2.07 × 10^6^	4.19	58.70
1	53.72	9.58	9.81	52.43
2	19.31	4.54	5.86	48.71
3	8.21	2.85	2.77	44.25
4	4.35	2.02	1.44	39.88
5	2.68	1.50	0.64	37.44
6	1.83	1.18	0.03	34.03
7	1.33	0.97	0.39	30.09
8	1.02	0.81	0.19	28.67
9	0.81	0.69	0.35	27.89
10	0.66	0.06	0.31	27.76
11	0.56	0.41	0.27	27.97
12	0.48	0.33	0.23	28.23
13	0.42	0.09	0.17	28.71
14	0.37	0.04	0.05	29.77

## 4 Conclusion

In this work, a comprehensive *ab initio* spectroscopic model for the nine lowest electronic states of PN is presented. A full set of potential energy, (transition) dipole moment, spin–orbit coupling, and electronic angular momenta coupling curves for these 9 electronic states was produced *ab initio* using the icMRCI + Q/ECP10MWB and icMRCI/aug-cc-pV5Z(-DK) methods. These curves were then processed *via* the Duo program to solve the fully-coupled nuclear-motion Schrödinger equation. Many of the results show satisfactory agreement with previous computational works, but there are certain differences in lifetimes and predicted spectra which indicate that further investigation backed by experimental data is needed to obtain a reliable spectroscopic model. In the next work we aim to tackle such differences, by producing an accurate, empirical line list for ^31^P^14^N for use in astrophysical spectroscopy of distant stars and exoplanets. In order to achieve this, the *ab initio* curves will be refined by fitting to the experimental data collected and processed *via* MARVEL methodology.^56^ Additionally non-adiabatic coupling effects, which are most important for the heavily perturbed *A*^1^Π state, will be included for finer accuracy. With PN being detected in multiple different interstellar media and phosphorous being a key element to life as we know it, PN is becoming a more important spectroscopic molecule. This work should provide an improved data quality for spectroscopic searches of PN in different astronomical environments, potentially leading to the detection of PN at ultraviolet wavelengths.

The supplementary materials in this paper contain our final spectroscopic model for PN in a form of a Duo input file, an ExoMol States file including lifetimes and a partition function file. This Duo file is ready to be directly used with the Duo program, see http://exomol.com/software/ allowing our results to be reproduced directly.

## Conflicts of interest

There are no conflicts to declare.

## Supplementary Material

CP-023-D1CP02537F-s001

CP-023-D1CP02537F-s002

CP-023-D1CP02537F-s003

CP-023-D1CP02537F-s004
